# Heart Failure Pathogenesis Elucidation and New Treatment Method Development

**DOI:** 10.31662/jmaj.2022-0106

**Published:** 2022-08-01

**Authors:** Mikako Katagiri, Shintaro Yamada, Manami Katoh, Toshiyuki Ko, Masamichi Ito, Issei Komuro

**Affiliations:** 1 Department of Cardiovascular Medicine, Graduate School of Medicine, the University of Tokyo, Tokyo, Japan; 2 Genome Science Laboratory, Research Center for Advanced Science and Technology, the University of Tokyo, Tokyo, Japan

**Keywords:** heart failure, cardiac hypertrophy, p53 signaling, DNA Damage, dilated cardiomyopathy, hypertrophic cardiomyopathy

## Abstract

Heart failure (HF) is a leading cause of death worldwide. In Japan, the number of HF patients has increased with its aging population, resulting in “HF pandemic.” HF is the final stage of various cardiovascular diseases, including valvular heart disease, ischemic heart disease, atrial fibrillation, and hypertension.

Cardiac hypertrophy is a compensatory response to increased workload and maintains cardiac function. Pressure overload due to mechanical stress causes cardiac hypertrophy, whereas continuous cardiac stress reduces wall thickness and consequently causes HF. Understanding the molecular mechanisms underlying this process is crucial to elucidate HF pathophysiology.

We demonstrated that ischemia and DNA damage are important in the progression of hypertrophy to HF. Genetic mutations associated with cardiomyopathy and prognosis has been identified. To realize precision medicines for HF, the underlying molecular mechanisms need to be elucidated. In this review, we introduce new paradigms for understanding HF pathophysiology discovered through basic research.

## 1. Introduction

Heart failure (HF) is a leading cause of death worldwide, and the number of HF patients continues to increase ^(1)^. It is estimated that there are currently approximately 1.2 million HF patients in Japan possibly due to the aging population. HF is a disease of the elderly, with approximately 20 patients per 100,000 people aged below 64 years but more than 10-fold patients aged above 65 years.

HF is the final stage of various cardiovascular diseases (CVDs). The causes are diverse, including valvular heart disease (2 million people in Japan), ischemic heart disease (approximately 800,000 people), atrial fibrillation (approximately 800,000 people), hypertension (approximately 43 million people), and congenital heart disease (approximately 400,000 people). The 4-year survival rate for HF is 55.8%, which is lower than that for cancer.

Being among the most rapidly aging societies in the world, Japan has an increasing number of HF patients, resulting in “HF pandemic,” and we have been searching for ways to overcome this problem. Thus, it is essential to conduct fundamental research to understand the process through which the heart acquires normal function, adaptively responds to stress, and then declines cardiac function.

We attempted to formulate research issues based on clinical questions collected through patient care, elucidate disease pathogenesis through basic research, translate the findings into clinical practice through translational research, and develop new approaches for the treatment of patients with diseases that have no established cure. We aimed to establish a virtuous cycle of clinical research for CVDs, including HF, whereby advanced medical care is provided to patients.

Here, we introduce new paradigms for understanding HF pathophysiology through basic research.

## 2. Cardiac Hypertrophy

### 2.1. Mechanism of cardiac hypertrophy

**Figure 1.**
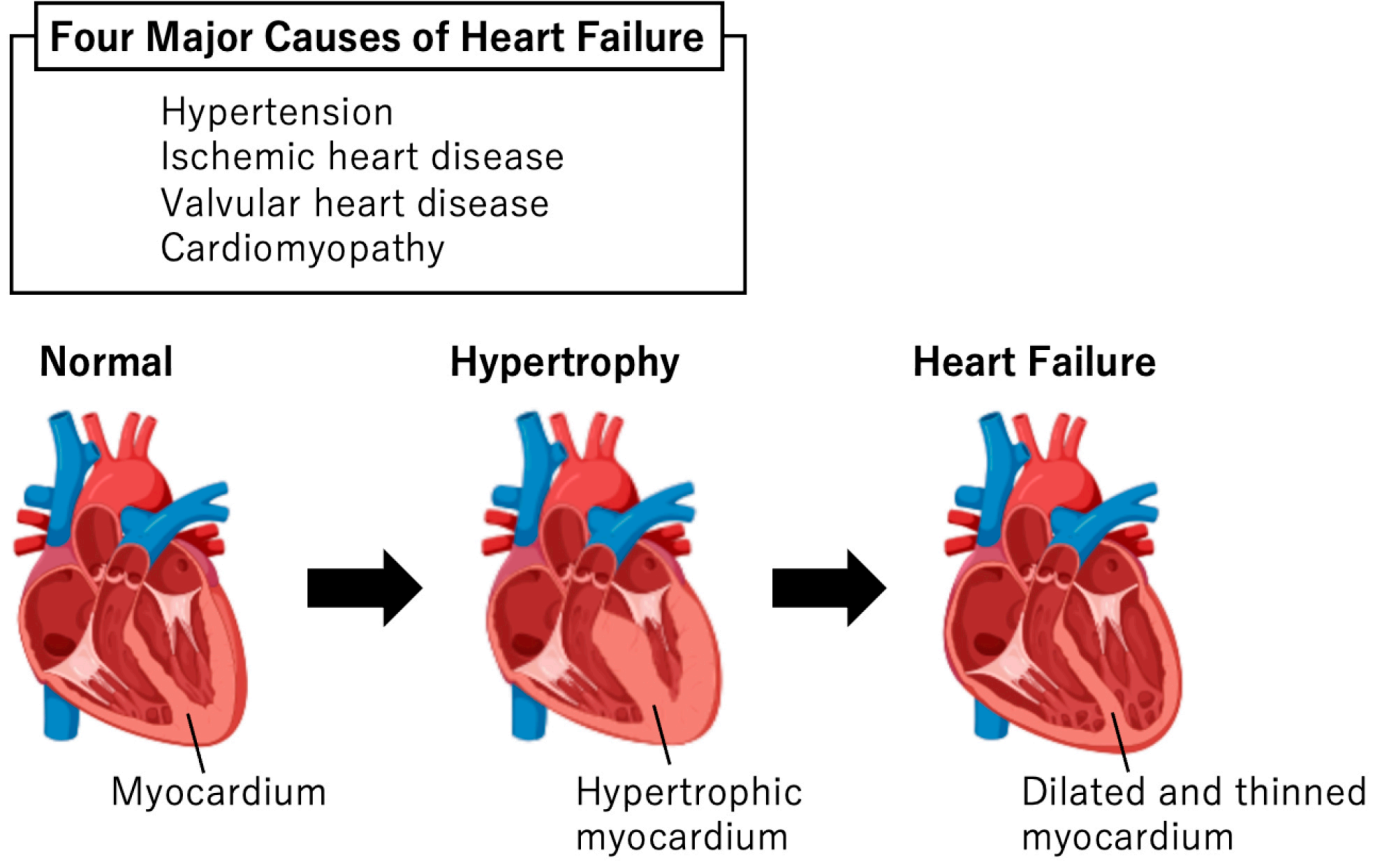
Four major causes and time course of heart failure (HF) Hypertension, ischemic heart disease, valvular disease, and cardiomyopathy are major HF causes. Cardiac stress causes left ventricular hypertrophy, and continuous stress reduces wall thickness, consequently causing HF.

Cardiac hypertrophy is an adaptive response to cardiac stress in various CVDs, such as hypertension, ischemic heart disease, valvular disease, and cardiomyopathy ^(2)^. Although left ventricular wall thickness initially increases to decrease wall stress based on Laplace’s law and maintains cardiac output, this compensation is finite ^(3)^. Continuous cardiac stress reduces wall thickness and consequently causes HF (Figure 1). Understanding the molecular mechanisms underlying cardiac hypertrophy is crucial to elucidate HF pathophysiology. Pressure or volume overload in CVDs causes excess mechanical stretching in cardiomyocytes, which directly regulates gene expression and triggers pathological cardiac hypertrophy.

### 2.2. Angiotensin II

Mechanical stretching initially activates angiotensin II type 1 (AT1) receptor and then activates extracellular signal-regulated kinase (ERK) ^(4)^, which subsequently phosphorylates Elk-1 that binds to the serum response element and activates *c-fos* transcription^(5)^. c-Fos forms a heterodimeric transcription factor, AP-1, which induces the transcription of several muscle-specific genes, including *Gata4* ^(6)^. Mechanical stretch in cultured rat neonatal cardiomyocytes changed the *c-fos* expression within 15 min after initiating the stretching stimulation ^(7)^. Overall, mechanical stress due to various CVDs directly regulates cardiac hypertrophy-associated gene transcription without humoral factor intervention.

## 3. Cardiac Development, Regeneration, and Aging

### 3.1. Cardiac homeobox transcription factor *Csx/Nkx2.5*

The heart develops from two cardiac progenitor cell pools: the first heart field and the second heart field ^(8)^. Multiple transcription factors play critical roles in heart development. *Nkx2.5*, also known as* Csx,* is a cardiac-specific transcription factor ^(9), (10)^ that appears both in the first and second heart fields and is essential in heart formation ^(11)^. Mutations in *NKX2.5* cause congenital heart diseases, including atrial septal defects, ventricular septal defects, and tetralogy of Fallot ^(12)^. *Nkx2.5* regulates cardiomyocyte proliferation in the developing heart ^(13)^. In addition, it works with other transcription factors, such as *Tbx5*,* Srf*, *Mef2c*, and *Gata4*, to promote cardiomyocyte differentiation ^(14), (15), (16), (17), (18)^.

### 3.2. Wnt/β-catenin signaling

Wnt signaling and insulin-like growth factor-binding protein 4 (IGFBP-4) are critical factors in cardiomyocyte differentiation. Wnt/β-catenin signaling activation during early embryoid body formation drives embryonic stem cell differentiation into cardiomyocytes ^(19)^. Contrarily, Wnt/β-catenin signaling activation during late embryoid body formation inhibits differentiation into cardiomyocytes ^(20)^. IGFBP-4 plays a crucial role in the Wnt/β-catenin pathway during heart development by inhibiting Wnt3A binding to its receptor and Wnt signaling activation and promoting differentiation into cardiomyocytes ^(21)^.

### 3.3. Humoral factors regulating aging

Senescence is a biological process resulting from homeostasis breakdown during aging ^(22)^. CVD significantly increases with age, and aging is a common and significant risk factor ^(23)^. One of the causes of senescence is declined tissue stem cell function. The environment surrounding organs, including signal transduction and humoral factors, play an essential role in maintaining tissue stem cell function ^(22)^. As aforementioned, one such environmental factor is Wnt signaling ^(24)^. It plays an important role in tissue stem cell maintenance by regulating cell proliferation, differentiation, and motility ^(25)^. Abnormal activation of Wnt signaling has also been implicated in cancer ^(26)^.

Rando et al. employed parabiosis surgery, in which the vasculature of a young and aged mouse was combined to share blood circulation to investigate the relationship between humoral factors and aging ^(27)^. The proliferative capacity of satellite cells, which are skeletal muscle tissue stem cells, is reduced, and their ability to regenerate skeletal muscle following an injury is disrupted in aged mice. Parabiosis surgery improved and decreased the tissue regenerative capacity and proliferative ability of satellite cells in aged and young mice, respectively, indicating the presence of senescence-accelerating substances in the serum of old mice. Such substances bind to the Wnt receptor Frizzled (Fz) and activate canonical Wnt signaling in a β-catenin-dependent manner ^(28)^. However, Wnt proteins are highly hydrophobic and difficult to transport through the bloodstream, indicating the presence of other aging-promoting and Wnt signaling-activating water-soluble molecules in the blood.

We found that the serum of HF mice activated Wnt signaling. Therefore, we considered the possibility that Wnt activators are involved in HF pathogenesis and identified them ^(29)^. A comprehensive analysis of Fz-binding proteins using serum from HF mice revealed that the complement molecule C1q bound to Fz increased in the serum of old and HF mice and cleaved the Wnt receptor LRP5/6 *via* C1r and C1s. C1q decreased the proliferative capacity of satellite cells *in vitro*, promoted fibroblast proliferation, and increased collagen production. The skeletal muscle regenerative capacity of aged mice was improved by C1q loss or C1s inhibitor administration, indicating that C1q activates Wnt signaling in the serum and promotes aging.

In summary, we proposed a new paradigm in which C1q-mediated Wnt activation induces aging in CVDs. The link between innate immunity and aging has a great potential for preventing and treating age-related diseases.

## 4. Heart Failure

Various factors including hemodynamic stress ^(30), (31)^, aging ^(32), (33)^, neurohumoral factors ^(34)^, developmental abnormalities ^(35)^, cell death ^(36)^, mitochondrial disorders ^(37)^, myocardial ischemia ^(38), (39)^, calcium regulation ^(40)^, catecholamine receptors ^(34), (41)^, inflammation ^(42), (43)^, metabolism ^(44), (45)^, and oxidative stress ^(43), (46)^ are involved in HF development.

### 4.1. Ischemia and p53 signaling

Several primary diseases, such as ischemic heart disease, valvular disease, and hypertension, cause HF, and some disease groups exhibit cardiac hypertrophy in response to stimuli before cardiac dysfunction. Cardiac hypertrophy is a compensatory response to increased workload and maintains cardiac function, which, if prolonged, can cause HF ^(3)^. To date, what happens during the progression of hypertrophy to HF is unknown.

We created mouse cardiac hypertrophy models *via* transverse aortic constriction (TAC), which causes temporary cardiac hypertrophy and cardiac dysfunction in succession ^(47)^. These mice exhibited adaptive cardiac hypertrophy to pressure overload until day 14, after which cardiac function gradually declined and fibrosis developed, leading to systolic dysfunction. The number of microvessels per myocyte in the heart increased during the hypertrophic phase, inhibiting angiogenesis-promoting factors, such as vascular endothelial growth factor (VEGF); angiopoietin-I suppressed cardiac hypertrophy, and worsened cardiac dysfunction. *Hif1a* expression enhanced from postoperative day 3 and then decreased during the HF phase. Because cardiomyocyte-specific *Hif1a* knockout reduced VEGF and microvessel expression, reduced *Hif1a* expression may be involved in HF phase induction. These results indicate that the hypoxic environment in the heart activates *Hif1a* to induce angiogenic factors during the compensatory phase, and the resulting adaptive hypertrophy maintains cardiac function. Contrarily, angiogenesis is inhibited during the decompensatory phase, and the blood flow supply cannot meet the demands of the myocardium, thus deteriorating cardiac function.

**Figure 2.**
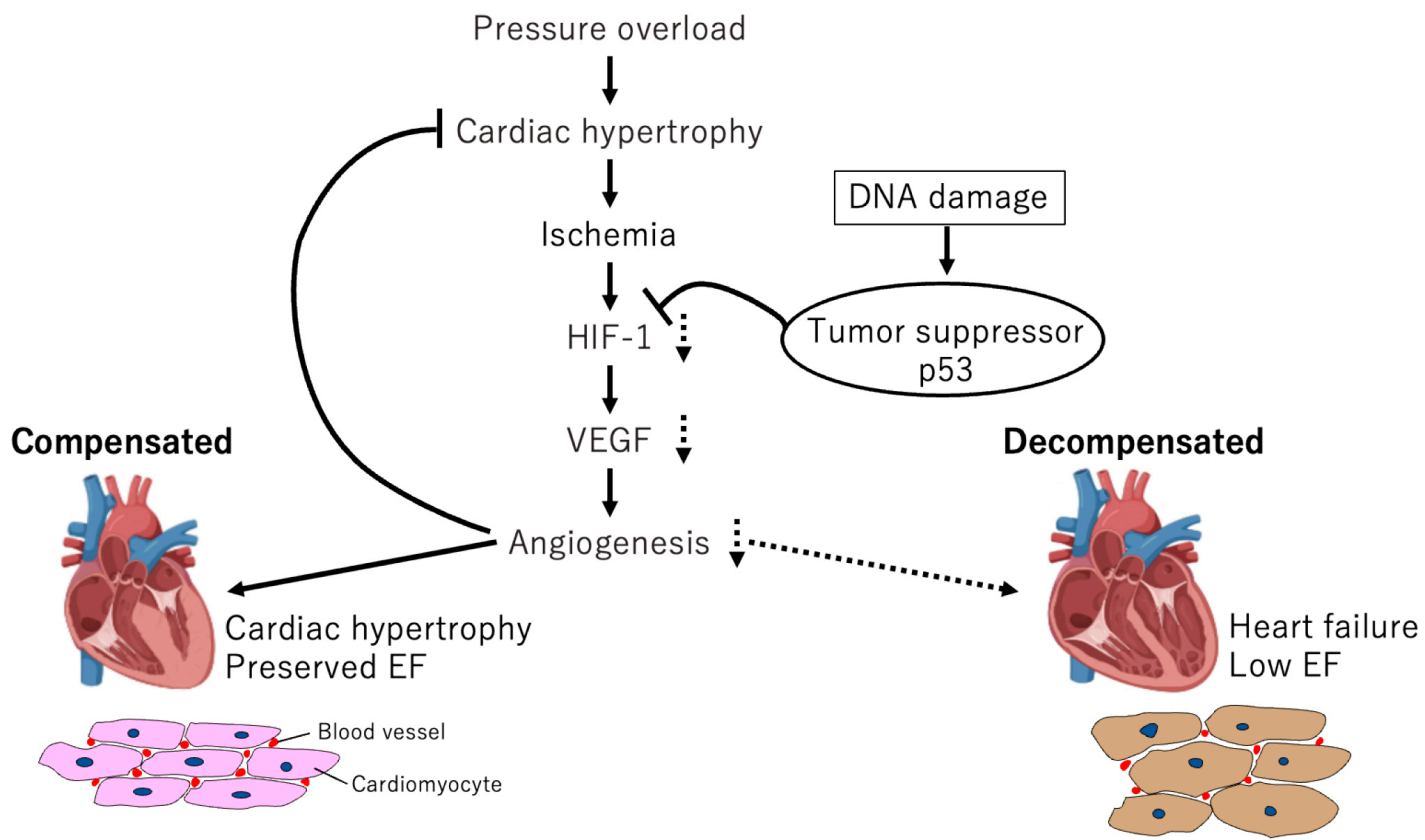
Molecular mechanisms for heart failure (HF) Cardiac hypertrophy is a compensatory response that increases workload and maintains cardiac function. Pressure overload due to mechanical stress causes cardiac hypertrophy, followed by ischemia. Ischemia increases *Hif1* expression, which induces VEGF and microvessel expression. Angiogenesis leads to compensated cardiac hypertrophy. The tumor suppressor p53 also impairs angiogenesis *via* *Hif1* activity inhibition. However, reduced *Hif1* expression may be involved in HF phase induction. VEGF, vascular endothelial growth factor; EF, ejection fraction.

The mechanism underlying this limitation of angiogenesis is unknown. Persistent hypoxia in cardiomyocytes during the chronic phase suggests that some factors promote Hif1a degradation in cardiomyocytes. p53, a known Hif-degrading factor ^(48)^, is activated in failing cardiomyocytes. p53 knockout mice following TAC surgery exhibited increased angiogenesis and Hif1a activity and suppressed cardiac function decline. This indicates that p53, a tumor suppressor, controls the turning point from compensation to HF through angiogenesis suppression in the heart, which suggests a new molecular mechanism for HF (Figure 2).

We further demonstrated this “ischemia-induced HF” clinically. Because peripheral blood mononuclear cell (PBMC) infusion promotes angiogenesis in ischemic tissue ^(49)^, we administered PBMC fractions to critical ischemic limb patients and analyzed their long-term outcomes. The results indicated improved ischemic limb symptoms in 70% patients and marked femoral amputation reduction. Myocardial perfusion SPECT imaging was also performed to evaluate myocardial ischemia before and after treatment. The degree of myocardial ischemia showed a significant decrease, and cardiac function showed a trend toward improvement after treatment, which was associated with peak plasma levels of VEGF, suggesting that PBMC infusion-induced angiogenic factors induced myocardial neovascularization and improved cardiac function by reducing ischemia ^(50)^.

### 4.2. DNA damage

Despite numerous basic studies outlining the mechanisms underlying cardiac hypertrophy, novel mechanisms remain to be identified, highlighting the complexity of this phenotype. Like in other diseases, oxidative stress is a well-known pathogenesis in HF development and progression^(46)^. The biological system of redox reactions may break out of its equilibrium state when the formation of reactive oxygen species overcomes antioxidant defense. This scenario favors the oxidation of biomolecules, such as proteins, lipids, and DNA, inside the cell, causing structural and functional damage and contributing to significant pathological outcomes. For decades, we have focused on the relationship between DNA damage and HF. Maintaining the correct genetic sequence is crucial for maintaining not only healthy cell division but also the function of differentiated cells, such as cardiomyocytes. Alterations in the genetic sequence, including single-strand breaks (SSBs) and double-strand breaks (DSBs), would activate the complex DNA surveillance machinery to recognize DNA damage, repair the breaks, or initiate cell death in case of excessive DNA damage. Using a murine pressure overload HF model, Higo et al. demonstrated DNA damage accumulation over time after pressure overload ^(51)^. Both SSBs ^(51)^ and DSBs ^(52)^ are induced by pressure overload and can be therapeutic targets for HF. Moreover, p53 signaling is activated during the development of maladaptive cardiac hypertrophy, which initiates cardiac remodeling-associated gene expression ^(53)^ and impairs angiogenesis through Hif-1 activity inhibition ^(47)^.

DNA damage is also an important factor in human heart disease. Although therapies for HF have decreased mortality rates, dilated cardiomyopathy (DCM) still has a poor prognosis and is the most prominent cause of heart transplantation in Japan. We have previously reported p53 signaling activation in the heart tissue of DCM patients ^(53)^. Using myocardial biopsy specimens obtained from DCM patients, we also demonstrated the utility of DNA damage marker staining for the prognostic prediction of medical therapy ^(54)^. Previous studies have demonstrated that *LMNA* mutations activate DNA damage response ^(55)^ and PARP1 ^(56), (57)^, followed by mitochondrial NAD^+^ consumption, which drive cardiomyocyte dysfunction and cardiomyopathy onset ^(56)^. Vignier et al. reported that cardiac dysfunction in DCM was ameliorated by supplementation with nicotinamide, an NAD^+^ precursor ^(57)^. Recently, Zhang et al. have reported the presence of DNA damage-associated dysmorphic nuclei and increased PARP1 activation in experimental and clinical atrial fibrillation ^(58)^. Their findings indicated that PARP1 inhibition, as well as NAD^+^ supplementation, may preserve atrial cardiomyocyte function in atrial fibrillation patients.

Despite several studies outlining the importance of DNA damage in various heart diseases, we have not fully elucidated the specific molecular mechanisms underlying DNA damage involved in HF development and progression. Although we have demonstrated the utility of evaluating DNA damage for the prognostic stratification of DCM patients, it remains unclear whether assessing DNA damage is also useful for other types of HF. Because artificial intelligence (AI) and machine learning are widely used in cardiovascular medicine ^(59), (60)^, they may also be helpful in detecting and evaluating DNA damage in HF patients. Overcoming technical limitations and a comprehensive and systematic understanding of DNA damage and HF can cause major breakthroughs in the future.

### 4.3. Genetic and environmental factors

The genetic background is also an important cause of HF. Technological innovations stimulate genomic research data collection and analysis, generating new knowledge and further biological hypotheses for validation in basic genomic research, particularly in animal experiments. In a genomic learning healthcare system, the application of novel genomic medical practice innovations based on this new knowledge will enable outcome data collection and analysis and help generate genomic knowledge and strategies to improve clinical care quality.

HF pathogenesis is primarily associated with genetic and environmental factors. Genetic factors include genomic mutations that cause abnormal gene expression and genetic polymorphisms that alter transcriptional factor activity, thus changing gene expression. Environmental factors include smoking, obesity, alcohol abuse, pregnancy, and drug treatment. Lifestyle-related diseases, such as diabetes, dyslipidemia, and hypertension, are associated with both genetic and environmental factors. With their coexistence, heart diseases, such as myocardial infarction, arrhythmia, and valvular heart disease, occur. Furthermore, the genetic background and environmental factors may cause HF ^(61)^.

### 4.4. Genomic variants associated with dilated cardiomyopathy

DCM is associated with genetic mutations, such as* Lamin A/C* (*LMNA*) and *Titin* (*TTN*), and characterized by ventricular enlargement as well as severe systolic dysfunction, which ultimately causes cardiac death ^(62)^. A panel sequencing analysis of 50 cardiomyopathy-causing genes in 120 DCM patients in Japan identified mutations in 65% cases, including *TTN* (25.6%) and *LMNA* (16.7%). In hypertrophic cardiomyopathy (HCM), mutations in genes, such as *myosin light chain 7* (*MYH7*) and *myosin binding protein C* (*MYBPC3*)*,* were found in almost half of the patients ^(62), (63)^. DCM patients with *TTN* truncating mutations had good prognosis after drug therapy. Contrarily, patients with *LMNA* mutations were associated with poor prognosis and tended to require heart transplantation. Thus, precision medicine for cardiomyopathy can be realized by considering the genetic information.

### 4.5. Genomic variants associated with CVDs

CVDs are “ultra-complex systems”; however, a comprehensive genetic analysis of large populations enables quantitative inference of the risk for diseases with complex biological backgrounds. Koyama et al. identified eight new susceptibility loci and rare Japanese-specific coronary artery disease (CAD) variants that increased severity and mortality using genome-wide association study (GWAS) in a Japanese population of 168,228 individuals. Furthermore, they derived a multifactorial risk score (PRS) predicting cardiovascular events by summing the risk of each SNP type through a meta-analysis of GWAS for CAD. The established PRS outperformed existing studies in predicting CAD outcomes, and the accuracy of prediction using this PRS exceeded that of judgments based on classical clinical risk factors. In future, such cross-sectional meta-analyses of GWAS data will accurately predict various diseases, including HF ^(64)^.

## 5. Vascular Regeneration

### 5.1. Peripheral arterial disease

Peripheral arterial disease is a general term for diseases that cause vascular circulatory failure of the lower extremities ^(65)^ and most commonly caused by atherosclerosis obliterans (ASO) and thromboangiitis obliterans (Buerger’s disease). ASO tends to occur in the abdominal and lower-extremity arteries of men aged >50 years. At least 10 million people in the USA have ASO ^(66)^. Buerger’s disease, which predominantly affects the peripheral arteries of male smokers aged 40-45 years, is common among Asians and Jews ^(67)^ and affects over 10,000 people in Japan ^(68)^. ASO is caused by arteriosclerosis, whereas the cause of Buerger’s disease is not well understood.

### 5.2. Therapeutic angiogenesis

We compared the effect of PBMC implantation from the bone marrow or peripheral blood and found that both induce sufficient neovascularization in hindlimb ischemia in mice ^(49)^. PBMCs were extracted using a blood component separator from ASO and Buerger’s disease patients, and they were implanted in the ischemic site. Evaluation of patient walking distance, pain at rest, ischemic ulcer, and ankle-brachial index (ABI) showed improvement in 70% of the patients. To investigate the mechanism of this therapy, we examined the factors associated with treatment response and concluded that elevation of growth factors in the plasma correlated well with the clinical outcome, and the number of implanted cells did not affect clinical efficacy ^(49), (50)^. Examination of the muscle specimen following cell transplantation revealed that the muscle cells produced interleukin (IL)-1β and suggested that stimulated muscle cells, not implanted PBMCs, produced angiogenic factors, thereby promoting neovascularization in ischemic tissues. IL-1β is a potent angiogenic cytokine that induces several angiogenic factors, including VEGF ^(69)^. Germani et al. reported similar results, showing that myotube regeneration produces VEGF ^(70)^. We believe that the mechanism of this treatment is that transplanted PBMCs promote muscle satellite cell proliferation and angiogenic factor production, which regenerate blood vessels and improve ischemia.

## 6. Future Prospects

CVD, as aforementioned, is an “ultra-complex system.” Therefore, further studies on genomics, single-cell biology, and spatial transcriptomics will play a key role in the investigation of these mechanisms.

Single-cell analysis is a new technique for detecting the future with one cell, making it possible to obtain detailed information. We established a method for single-cell RNA-seq analysis of the heart and analyzed tissue samples from mouse HF models and HF patients ^(53)^. We found that both mouse and human myocardium divide into compensated and failing myocardium upon loading and that DNA damage and p53 signaling induce failing myocardium.

We believe that integrating big datasets, tissue omics information such as genomes, and vast clinical information using AI and machine learning ^(71), (72)^ will provide better understanding of CVD pathogenesis and help develop novel therapeutic approaches.

## Article Information

This article is based on the study, which received the Medical Award of The Japan Medical Association in 2021.

### Conflicts of Interest

None

### Sources of Funding

This work was supported by grants from a Grant-in-Aid for Young Scientists (to T.K. M.I., and Ma.K.), the Japan Foundation for Applied Enzymology (to S.Y. and T.K.), the SENSHIN Medical Research Foundation (to T.K.), MSD Life Science Foundation (to T.K.).

### Author Contributions

Mi.K. took the lead in drafting the manuscript. Mi.K., S.Y., Ma.K., T.K., and M.I. reviewed the literature and drafted the initial manuscript. I.K. supervised the writing of this work. All authors have read and approved the final vision of the manuscript.

## References

[1] GBD Disease and injury incidence and prevalence collaborators. Global, regional, and national incidence, prevalence, and years lived with disability for 354 diseases and injuries for 195 countries and territories, 1990-2017: a systematic analysis for the Global Burden of Disease Study 2017. Lancet. 2018;392(10159):1789-858. 30496104 10.1016/S0140-6736(18)32279-7 PMC6227754

[2] Frey N, Katus HA, Olson EN, et al. Hypertrophy of the heart: a new therapeutic target? Circulation. 2004;109(13):1580-9. 15066961 10.1161/01.CIR.0000120390.68287.BB

[3] Nakamura M, Sadoshima J. Mechanisms of physiological and pathological cardiac hypertrophy. Nat Rev Cardiol. 2018;15(7):387-407. 29674714 10.1038/s41569-018-0007-y

[4] Zou Y, Akazawa H, Qin Y, et al. Mechanical stress activates angiotensin II type 1 receptor without the involvement of angiotensin II. Nat Cell Biol. 2004;6(6):499-506. 15146194 10.1038/ncb1137

[5] Babu GJ, Lalli MJ, Sussman MA, et al. Phosphorylation of elk-1 by MEK/ERK pathway is necessary for c-fos gene activation during cardiac myocyte hypertrophy. J Mol Cell Cardiol. 2000;32(8):1447-57. 10900171 10.1006/jmcc.2000.1185

[6] Herzig TC, Jobe SM, Aoki H, et al. Angiotensin II type1a receptor gene expression in the heart: AP-1 and GATA-4 participate in the response to pressure overload. Proc Natl Acad Sci U S A. 1997;94(14):7543-8. 9207128 10.1073/pnas.94.14.7543 PMC23858

[7] Kurabayashi M, Komuro I, Shibasaki Y, et al. Functional identification of the transcriptional regulatory elements within the promoter region of the human ventricular myosin alkali light chain gene. J Biol Chem. 1990;265(31):19271-8. 1699944

[8] Srivastava D. Making or breaking the heart: from lineage determination to morphogenesis. Cell. 2006;126(6):1037-48. 16990131 10.1016/j.cell.2006.09.003

[9] Komuro I, Izumo S. Csx: a murine homeobox-containing gene specifically expressed in the developing heart. Proc Natl Acad Sci U S A. 1993;90(17):8145-9. 7690144 10.1073/pnas.90.17.8145 PMC47305

[10] Akazawa H, Komuro I. Cardiac transcription factor Csx/Nkx2-5: its role in cardiac development and diseases. Pharmacol Ther. 2005;107(2):252-68. 15925411 10.1016/j.pharmthera.2005.03.005

[11] Zhang L, Nomura-Kitabayashi A, Sultana N, et al. Mesodermal Nkx2.5 is necessary and sufficient for early second heart field development. Dev Biol. 2014;390(1):68-79. 24613616 10.1016/j.ydbio.2014.02.023 PMC4461860

[12] Kasahara H, Lee B, Schott JJ, et al. Loss of function and inhibitory effects of human CSX/NKX2.5 homeoprotein mutations associated with congenital heart disease. J Clin Invest. 2000;106(2):299-308. 10903346 10.1172/JCI9860 PMC314312

[13] Horton AJ, Brooker J, Streitfeld WS, et al. Nkx2-5 second heart field target gene Ccdc117 regulates DNA metabolism and proliferation. Sci Rep. 2019;9(1):1738. 30742009 10.1038/s41598-019-39078-5 PMC6370788

[14] Chen CY, Croissant J, Majesky M, et al. Activation of the cardiac α-actin promoter depends upon serum response factor, Tinman homologue, Nkx-2.5, and intact serum response elements. Dev Genet. 1996;19(2):119-30. 8900044 10.1002/(SICI)1520-6408(1996)19:2<119::AID-DVG3>3.0.CO;2-C

[15] Skerjanc IS, Petropoulos H, Ridgeway AG, et al. Myocyte enhancer factor 2C and Nkx2-5 up-regulate each other's expression and initiate cardiomyogenesis in P19 cells. J Biol Chem. 1998;273(52):34904-10. 9857019 10.1074/jbc.273.52.34904

[16] Monzen K, Shiojima I, Hiroi Y, et al. Bone morphogenetic proteins induce cardiomyocyte differentiation through the mitogen-activated protein kinase kinase kinase TAK1 and cardiac transcription factors Csx/Nkx-2.5 and GATA-4. Mol Cell Biol. 1999;19(10):7096-105. 10490646 10.1128/mcb.19.10.7096 PMC84704

[17] Shiojima I, Komuro I, Oka T, et al. Context-dependent transcriptional cooperation mediated by cardiac transcription factors Csx/Nkx-2.5 and GATA-4. J Biol Chem. 1999;274(12):8231-9. 10075728 10.1074/jbc.274.12.8231

[18] Hiroi Y, Kudoh S, Monzen K, et al. Tbx5 associates with Nkx2-5 and synergistically promotes cardiomyocyte differentiation. Nat Genet. 2001;28(3):276-80. 11431700 10.1038/90123

[19] Paige SL, Osugi T, Afanasiev OK, et al. Endogenous Wnt/beta-catenin signaling is required for cardiac differentiation in human embryonic stem cells. PLOS ONE. 2010;5(6):e11134. 20559569 10.1371/journal.pone.0011134 PMC2886114

[20] Naito AT, Shiojima I, Akazawa H, et al. Developmental stage-specific biphasic roles of Wnt/beta-catenin signaling in cardiomyogenesis and hematopoiesis. Proc Natl Acad Sci U S A. 2006;103(52):19812-7. 17170140 10.1073/pnas.0605768103 PMC1750922

[21] Zhu W, Shiojima I, Ito Y, et al. IGFBP-4 is an inhibitor of canonical Wnt signalling required for cardiogenesis. Nature. 2008;454(7202):345-9. 18528331 10.1038/nature07027

[22] Di Micco R, Krizhanovsky V, Baker D, et al. Cellular senescence in ageing: from mechanisms to therapeutic opportunities. Nat Rev Mol Cell Biol. 2021;22(2):75-95. 33328614 10.1038/s41580-020-00314-w PMC8344376

[23] Fyhrquist F, Saijonmaa O, Strandberg T. The roles of senescence and telomere shortening in cardiovascular disease. Nat Rev Cardiol. 2013;10(5):274-83. 23478256 10.1038/nrcardio.2013.30

[24] Hu HH, Cao G, Wu XQ, et al. Wnt signaling pathway in aging-related tissue fibrosis and therapies. Ageing Res Rev. 2020;60:101063. 32272170 10.1016/j.arr.2020.101063

[25] Reya T, Duncan AW, Ailles L, et al. A role for Wnt signalling in self-renewal of haematopoietic stem cells. Nature. 2003;423(6938):409-14. 12717450 10.1038/nature01593

[26] Zhan T, Rindtorff N, Boutros M. Wnt signaling in cancer. Oncogene. 2017;36(11):1461-73. 27617575 10.1038/onc.2016.304 PMC5357762

[27] Conboy IM, Conboy MJ, Wagers AJ, et al. Rejuvenation of aged progenitor cells by exposure to a young systemic environment. Nature. 2005;433(7027):760-4. 15716955 10.1038/nature03260

[28] Brack AS, Conboy MJ, Roy S, et al. Increased Wnt signaling during aging alters muscle stem cell fate and increases fibrosis. Science. 2007;317(5839):807-10. 17690295 10.1126/science.1144090

[29] Naito AT, Sumida T, Nomura S, et al. Complement C1q activates canonical Wnt signaling and promotes aging-related phenotypes. Cell. 2012;149(6):1298-313. 22682250 10.1016/j.cell.2012.03.047 PMC3529917

[30] Komuro I, Yazaki Y. Control of cardiac gene expression by mechanical stress. Annu Rev Physiol. 1993;55:55-75. 8466185 10.1146/annurev.ph.55.030193.000415

[31] Satoh M, Nomura S, Harada M, et al. High-throughput single-molecule RNA imaging analysis reveals heterogeneous responses of cardiomyocytes to hemodynamic overload. J Mol Cell Cardiol. 2019;128:77-89. 30611794 10.1016/j.yjmcc.2018.12.018

[32] Morita H, Komuro I. Heart failure as an aging-related phenotype. Int Heart J. 2018;59(1):6-13. 29332923 10.1536/ihj.17-519

[33] Daneshgar N, Rabinovitch PS, Dai DF. TOR signaling pathway in cardiac aging and heart failure. Biomolecules. 2021;11(2):168. 33513917 10.3390/biom11020168 PMC7911348

[34] von Lueder TG, Kotecha D, Atar D, et al. Neurohormonal blockade in heart failure. Card Fail Rev. 2017;3(1):19-24. 28785471 10.15420/cfr.2016:22:2 PMC5494151

[35] Wang T, Chen L, Yang T, et al. Congenital heart disease and risk of cardiovascular disease: a meta-analysis of cohort studies. J Am Heart Assoc. 2019;8(10):e012030. 31070503 10.1161/JAHA.119.012030 PMC6585327

[36] Abbate A, Bussani R, Biondi-Zoccai GG, et al. Persistent infarct-related artery occlusion is associated with an increased myocardial apoptosis at postmortem examination in humans late after an acute myocardial infarction. Circulation. 2002;106(9):1051-4. 12196327 10.1161/01.cir.0000030936.97158.c4

[37] Mazzaccara C, Mirra B, Barretta F, et al. Molecular epidemiology of mitochondrial cardiomyopathy: a search among mitochondrial and nuclear genes. Int J Mol Sci. 2021;22(11):5742. 34072184 10.3390/ijms22115742 PMC8197938

[38] Hasegawa H, Takano H, Iwanaga K, et al. Cardioprotective effects of granulocyte colony-stimulating factor in swine with chronic myocardial ischemia. J Am Coll Cardiol. 2006;47(4):842-9. 16487854 10.1016/j.jacc.2005.09.048

[39] Jenča D, Melenovský V, Stehlik J, et al. Heart failure after myocardial infarction: incidence and predictors. ESC Heart Fail. 2021;8(1):222-37. 33319509 10.1002/ehf2.13144 PMC7835562

[40] Kamimura D, Ohtani T, Sakata Y, et al. Ca2+ entry mode of Na+/Ca2+ exchanger as a new therapeutic target for heart failure with preserved ejection fraction. Eur Heart J. 2012;33(11):1408-16. 21490055 10.1093/eurheartj/ehr106

[41] Cleland JGF, Bunting KV, Flather MD, et al. Beta-blockers for heart failure with reduced, mid-range, and preserved ejection fraction: an individual patient-level analysis of double-blind randomized trials. Eur Heart J. 2018;39(1):26-35. 29040525 10.1093/eurheartj/ehx564 PMC5837435

[42] Dorsheimer L, Assmus B, Rasper T, et al. Association of mutations contributing to clonal hematopoiesis with prognosis in chronic ischemic heart failure. JAMA Cardiol. 2019;4(1):25-33. 30566180 10.1001/jamacardio.2018.3965 PMC6439691

[43] Toyoda S, Haruyama A, Inami S, et al. Effects of carvedilol vs bisoprolol on inflammation and oxidative stress in patients with chronic heart failure. J Cardiol. 2020;75(2):140-7. 31444140 10.1016/j.jjcc.2019.07.011

[44] Minamino T, Orimo M, Shimizu I, et al. A crucial role for adipose tissue p53 in the regulation of insulin resistance. Nat Med. 2009;15(9):1082-7. 19718037 10.1038/nm.2014

[45] Aoshima C, Fujimoto S, Kudo A, et al. Clinical significance of (123)I-BMIPP washout rate in patients with uncertain chronic heart failure. Eur J Nucl Med Mol Imaging. Forthcoming 2022. 10.1007/s00259-022-05749-1 35298692

[46] van der Pol A, van Gilst WH, Voors AA, et al. Treating oxidative stress in heart failure: past, present and future. Eur J Heart Fail. 2019;21(4):425-35. 30338885 10.1002/ejhf.1320 PMC6607515

[47] Sano M, Minamino T, Toko H, et al. p53-induced inhibition of Hif-1 causes cardiac dysfunction during pressure overload. Nature. 2007;446(7134):444-8. 17334357 10.1038/nature05602

[48] Ravi R, Mookerjee B, Bhujwalla ZM, et al. Regulation of tumor angiogenesis by p53-induced degradation of hypoxia-inducible factor 1alpha. Genes Dev. 2000;14(1):34-44. 10640274 PMC316350

[49] Tateno K, Minamino T, Toko H, et al. Critical roles of muscle-secreted angiogenic factors in therapeutic neovascularization. Circ Res. 2006;98(9):1194-202. 16574905 10.1161/01.RES.0000219901.13974.15

[50] Moriya J, Minamino T, Tateno K, et al. Long-term outcome of therapeutic neovascularization using peripheral blood mononuclear cells for limb ischemia. Circ Cardiovasc Interv. 2009;2(3):245-54. 20031722 10.1161/CIRCINTERVENTIONS.108.799361

[51] Higo T, Naito AT, Sumida T, et al. DNA single-strand break-induced DNA damage response causes heart failure. Nat Commun. 2017;8:15104. 28436431 10.1038/ncomms15104 PMC5413978

[52] Nakada Y, Nhi Nguyen NU, Xiao F, et al. DNA damage response mediates pressure overload-induced cardiomyocyte hypertrophy. Circulation. 2019;139(9):1237-9. 30802166 10.1161/CIRCULATIONAHA.118.034822 PMC6467068

[53] Nomura S, Satoh M, Fujita T, et al. Cardiomyocyte gene programs encoding morphological and functional signatures in cardiac hypertrophy and failure. Nat Commun. 2018;9(1):4435. 30375404 10.1038/s41467-018-06639-7 PMC6207673

[54] Ko T, Fujita K, Nomura S, et al. Quantification of DNA damage in heart tissue as a novel prediction tool for therapeutic prognosis of patients with dilated cardiomyopathy. JACC Basic Transl Sci. 2019;4(6):670-80. 31709317 10.1016/j.jacbts.2019.05.010 PMC6834953

[55] Chen SN, Lombardi R, Karmouch J, et al. DNA damage response/TP53 pathway is activated and contributes to the pathogenesis of dilated cardiomyopathy associated with LMNA (Lamin A/C) mutations. Circ Res. 2019;124(6):856-73. 30696354 10.1161/CIRCRESAHA.118.314238 PMC6460911

[56] Diguet N, Trammell SAJ, Tannous C, et al. Nicotinamide riboside preserves cardiac function in a mouse model of dilated cardiomyopathy. Circulation. 2018;137(21):2256-73. 29217642 10.1161/CIRCULATIONAHA.116.026099 PMC6954688

[57] Vignier N, Chatzifrangkeskou M, Morales Rodriguez B, et al. Rescue of biosynthesis of nicotinamide adenine dinucleotide protects the heart in cardiomyopathy caused by lamin A/C gene mutation. Hum Mol Genet. 2018;27(22):3870-80. 30053027 10.1093/hmg/ddy278

[58] Zhang D, Hu X, Li J, et al. DNA damage-induced PARP1 activation confers cardiomyocyte dysfunction through NAD^+^ depletion in experimental atrial fibrillation. Nat Commun. 2019;10(1):1307. 30898999 10.1038/s41467-019-09014-2 PMC6428932

[59] Shu S, Ren J, Song J. Clinical application of machine learning-based artificial intelligence in the diagnosis, prediction, and classification of cardiovascular diseases. Circ J. 2021;85(9):1416-25. 33883384 10.1253/circj.CJ-20-1121

[60] Matsumoto T, Kodera S, Shinohara H, et al. Diagnosing heart failure from chest X-ray images using deep learning. Int Heart J. 2020;61(4):781-6. 32684597 10.1536/ihj.19-714

[61] Zhang YB, Pan XF, Chen J, et al. Combined lifestyle factors, all-cause mortality and cardiovascular disease: a systematic review and meta-analysis of prospective cohort studies. J Epidemiol Community Health. 2021;75(1):92-9. 32892156 10.1136/jech-2020-214050

[62] Tobita T, Nomura S, Fujita T, et al. Genetic basis of cardiomyopathy and the genotypes involved in prognosis and left ventricular reverse remodeling. Sci Rep. 2018;8(1):1998. 29386531 10.1038/s41598-018-20114-9 PMC5792481

[63] Tobita T, Nomura S, Morita H, et al. Identification of MYLK3 mutations in familial dilated cardiomyopathy. Sci Rep. 2017;7(1):17495. 29235529 10.1038/s41598-017-17769-1 PMC5727479

[64] Koyama S, Ito K, Terao C, et al. Population-specific and trans-ancestry genome-wide analyses identify distinct and shared genetic risk loci for coronary artery disease. Nat Genet. 2020;52(11):1169-77. 33020668 10.1038/s41588-020-0705-3

[65] Hirsch AT, Haskal ZJ, Hertzer NR, et al. ACC/AHA 2005 Practice Guidelines for the management of patients with peripheral arterial disease (lower extremity, renal, mesenteric, and abdominal aortic): a collaborative report from the American Association for Vascular Surgery/Society for Vascular Surgery, Society for Cardiovascular Angiography and Interventions, Society for Vascular Medicine and Biology, Society of Interventional Radiology, and the ACC/AHA Task Force on Practice Guidelines (Writing Committee to Develop Guidelines for the Management of Patients With Peripheral Arterial Disease): endorsed by the American Association of Cardiovascular and Pulmonary Rehabilitation; National Heart, Lung, and Blood Institute; Society for Vascular Nursing; TransAtlantic Inter-Society Consensus; and Vascular Disease Foundation. Circulation. 2006;113(11):e463-654. 16549646 10.1161/CIRCULATIONAHA.106.174526

[66] Annex BH, Cooke JP. New directions in therapeutic angiogenesis and arteriogenesis in peripheral arterial disease. Circ Res. 2021;128(12):1944-57. 34110899 10.1161/CIRCRESAHA.121.318266 PMC8538391

[67] Arkkila PE. Thromboangiitis obliterans (Buerger's disease). Orphanet J Rare Dis. 2006;1:14. 16722538 10.1186/1750-1172-1-14 PMC1523324

[68] Watanabe Y, Miyata T, Shigematsu K, et al. Current trends in epidemiology and clinical features of thromboangiitis obliterans in Japan - A nationwide survey using the medical support system database. Circ J. 2020;84(10):1786-96. 32879220 10.1253/circj.CJ-19-1165

[69] El Awad B, Kreft B, Wolber EM, et al. Hypoxia and interleukin-1beta stimulate vascular endothelial growth factor production in human proximal tubular cells. Kidney Int. 2000;58(1):43-50. 10886548 10.1046/j.1523-1755.2000.00139.x

[70] Germani A, Di Carlo A, Mangoni A, et al. Vascular endothelial growth factor modulates skeletal myoblast function. Am J Pathol. 2003;163(4):1417-28. 14507649 10.1016/S0002-9440(10)63499-2 PMC1868307

[71] Ambale-Venkatesh B, Yang X, Wu CO, et al. Cardiovascular event prediction by machine learning: the multi-ethnic study of atherosclerosis. Circ Res. 2017;121(9):1092-101. 28794054 10.1161/CIRCRESAHA.117.311312 PMC5640485

[72] Kagiyama N, Piccirilli M, Yanamala N, et al. Machine learning assessment of left ventricular diastolic function based on electrocardiographic features. J Am Coll Cardiol. 2020;76(8):930-41. 32819467 10.1016/j.jacc.2020.06.061

